# Surface Ice Detection Using Hyperspectral Imaging and Machine Learning

**DOI:** 10.3390/s25144322

**Published:** 2025-07-10

**Authors:** Steve Vanlanduit, Arnaud De Vooght, Thomas De Kerf

**Affiliations:** InViLab Research Group, Department of Electromechanical Engineering, Faculty of Applied Engineering, University of Antwerp, Groenenborgerlaan 171, 2020 Antwerpen, Belgiumthomas.dekerf@uantwerpen.be (T.D.K.)

**Keywords:** hyperspectral imaging, ice detection, machine learning, wind turbine monitoring, random forest, support vector machine (SVM)

## Abstract

**Highlights:**

**What are the main findings?**

- Hyperspectral imaging combined with machine learning enables accurate surface ice detection.
- Support Vector Machine (SVM) and Random Forest (RF) classifiers were evaluated on real hyperspectral datasets.
- The models generalize well across coated and uncoated surfaces, including challenging dark coatings.
- Spectral band reduction was analyzed, revealing trade-offs between classification accuracy and computational efficiency.
- A multiclass classification approach was introduced to differentiate between rime and glaze ice.

**What is the implication of the main finding?**

- Demonstrates the feasibility of non-contact, hyperspectral-based methods for automated ice detection.
- Validates robustness of machine learning models across diverse material surfaces.
- Supports the development of efficient, real-time ice detection systems through reduced spectral dimensionality.
- Enhances safety applications by enabling discrimination between different ice types.
- Provides a foundation for integrating hyperspectral AI systems in energy, transportation and infrastructure monitoring.

**Abstract:**

Ice formation on critical infrastructure such as wind turbine blades can lead to severe performance degradation and safety hazards. This study investigates the use of hyperspectral imaging (HSI) combined with machine learning to detect and classify ice on various coated and uncoated surfaces. Hyperspectral reflectance data were acquired using a push-broom HSI system under controlled laboratory conditions, with ice and rime ice generated using a thermoelectric cooling setup. Support Vector Machine (SVM) and Random Forest (RF) classifiers were trained on uncoated aluminum samples and evaluated on surfaces with different coatings to assess model generalization. Both models achieved high classification accuracy, though performance declined on black-coated surfaces due to increased absorbance by the coating. The study further examined the impact of spectral band reduction to simulate different sensor types (e.g., NIR vs. SWIR), revealing that model performance is sensitive to wavelength range, with SVM performing optimally in a reduced band set and RF benefiting from the full spectral range. A multiclass classification approach using RF successfully distinguished between glaze and rime ice, offering insights into more targeted mitigation strategies. The results confirm the potential of HSI and machine learning as robust tools for surface ice monitoring in safety-critical environments.

## 1. Introduction

Ice accretion on exposed surfaces poses significant safety and performance challenges across a range of sectors, including renewable energy, aviation, transportation, and telecommunications. Wind turbines, in particular, are vulnerable to ice formation on their blades, which can lead to power losses, increased mechanical stress, and safety risks due to ice shedding [1,2]. Accurate and early detection of surface ice is therefore critical for ensuring operational efficiency and structural integrity [3].

Traditional ice detection techniques, such as thermal sensing [4], vibration analysis [5], or visual inspections, often suffer from limitations in sensitivity, range, and robustness under varying environmental conditions [6]. Recent advances in optical sensing and machine learning have opened new possibilities for more effective monitoring of ice accretion, especially in safety-critical environments. Several authors use thermal imaging to detect icing conditions [7,8]. While thermal imaging can be useful when a clear thermal gradient exists, it becomes unreliable when ice and substrate temperatures are similar or when environmental factors mask subtle thermal differences. This makes it unsuitable as a standalone method for robust icing detection on wind turbine blades in many real-world scenarios.

Hyperspectral imaging (HSI) is an emerging technique that combines spectral and spatial information to capture detailed material signatures across a wide range of wavelengths. Its ability to distinguish between subtle differences in surface composition makes it a promising candidate for detecting ice under diverse conditions and coatings. When paired with modern machine learning algorithms, hyperspectral imaging enables automated and scalable solutions for surface state classification [9].

Hyperspectral image classification has become an indispensable tool across diverse domains such as agriculture, geology, environmental monitoring, and medical diagnostics. While this study focuses on ice detection and classification, numerous state-of-the-art algorithms have been developed for other applications, offering important context for the selection of machine learning models. Traditional methods like Support Vector Machines (SVMs), Random Forests (RFs), and Maximum Likelihood Classification (MLC) remain popular due to their robustness and interpretability. For instance, in agricultural applications, SVM and RF have achieved classification accuracies exceeding 90% for tasks such as crop type identification and vegetation health assessment [10,11,12]. In geological mapping, Kumar et al. [13] and Rani et al. [14] showed that PCA followed by SVM or MLC provided accurate mineral classification with overall accuracies up to 97%. Environmental studies have also applied SVM and MLC for monitoring algal blooms and water quality, achieving performance ranging from 60% to 84% (see Eisenhardt et al. [15]).

More recent developments in deep learning have led to models that can exploit both spatial and spectral information in hyperspectral cubes. Convolutional Neural Networks (CNNs) are widely used due to their ability to learn localized features, while hybrid approaches such as CNN-Transformer models have been proposed for tasks requiring both global and local attention mechanisms [11]. Autoencoders and generative models are increasingly used for unsupervised classification and anomaly detection, such as the Memory-Augmented Autoencoder with Adaptive Reconstruction developed by Zhang et al. [16], which suppresses known background signatures while enhancing the detection of anomalous spectral patterns.

In the field of automated architecture design, Neural Architecture Search (NAS) frameworks are being used to discover optimal models for hyperspectral classification. The Triple-Unit NAS network proposed by Xie et al. [17] integrates spectral, spatial, and contextual learning blocks, significantly outperforming conventional CNNs on benchmark datasets. Similarly, Shah et al. [18] introduced an explainable attention-based deep network for the classification of plant species, integrating interpretable saliency maps to highlight informative spectral bands.

Beyond classification, advanced ensemble learning methods such as AdaBoost, stacking, and voting classifiers are also gaining traction. Asad et al. [19] (2021) compared various ensemble models for hyperspectral crop classification, highlighting trade-offs between interpretability, computational efficiency, and accuracy. For applications involving spectral ambiguity or overlapping classes, hybrid models that combine deep learning with rule-based systems are also emerging.

This paper investigates the use of hyperspectral imaging and supervised machine learning to detect different types of ice (rime and glaze ice) on coated and uncoated surfaces. A controlled experimental setup was developed to capture hyperspectral reflectance data under both icy and non-icy conditions. Multiple classification algorithms are trained and evaluated to assess their accuracy and robustness. Furthermore, spectral band reduction is performed to evaluate how changes in wavelength range, corresponding to the use of different types of cameras, such as those operating in the near-infrared (NIR) or short-wave infrared (SWIR) domains, affect detection performance.

## 2. Related Work

Ice detection on critical surfaces such as roads, aircraft, and wind turbine blades has long been a focus of research due to the significant safety and performance risks associated with undetected icing. Conventional techniques, ranging from thermal sensing and vibration sensing to visual inspection, face challenges in sensitivity, robustness, and suitability across diverse surface conditions. This has motivated the exploration of optical methods, particularly hyperspectral imaging (HSI).

In road surface applications, HSI has demonstrated high accuracy in differentiating between dry, wet, and icy conditions. For example, Nakauchi et al. employed bandpass-filtered spectral reflectance data to classify surface states of asphalt, achieving classification accuracies above 90% [20]. Similarly, Bhattacharyya et al. [21] developed a deep learning-based pipeline for black ice classification on asphalt, illustrating the effectiveness of spectral features in distinguishing ice-covered surfaces from bare pavement, even under visually ambiguous conditions.

In the domain of wind turbine monitoring, the application of HSI remains comparatively less mature but promising. Rizk et al. explored the use of hyperspectral imaging to detect both damage and ice on turbine blades [22,23]. Although quantitative performance metrics were not fully detailed, their preliminary results indicated the feasibility of HSI in this application. Moreover, Gómez Muñoz et al. [4] demonstrated that infrared thermography could detect ice on turbine blades but noted that its reliability decreases when the temperature difference between the ice and the blade surface is minimal, limiting its effectiveness under steady-state or uniformly cold conditions.

Aircraft de-icing is another area where HSI has shown potential. Zhang et al. recently reported a methodology to estimate ice thickness using spectral signatures, achieving an error margin of approximately 2 mm [24]. Their findings underscore the possibility of using hyperspectral data not only for binary classification (ice vs. no ice) but also for continuous variable estimation, such as thickness or roughness.

From a methodological perspective, recent studies have shifted toward the integration of machine learning and deep learning techniques to analyze hyperspectral data. Random Forests (RFs), Support Vector Machines (SVMs), and Convolutional Neural Networks (CNNs) are among the most widely used classifiers. Bhattacharyya et al. [21] and Musci et al. [25] have shown that deep architectures can outperform classical methods, especially in scenarios with limited spectral resolution or subtle spectral differences between ice and background materials, where traditional feature-based approaches may struggle.

Despite these advances, several gaps remain. Many studies rely on road surfaces, with limited investigation into metallic or coated materials. More critically, the influence of surface coatings, which may significantly alter spectral reflectance, has not been systematically studied in the context of ice detection. This presents a challenge for practical applications in aviation and renewable energy, where protective coatings are commonly applied to structural surfaces.

The present study addresses this gap by conducting controlled hyperspectral imaging experiments on both coated and uncoated aluminum surfaces. By evaluating multiple machine learning classifiers and assessing the impact of band selection, our work provides new insights into the robustness and generalizability of HSI-based ice detection across different material conditions.

## 3. Materials and Methods

### 3.1. Experimental Setup

**Figure 1:**
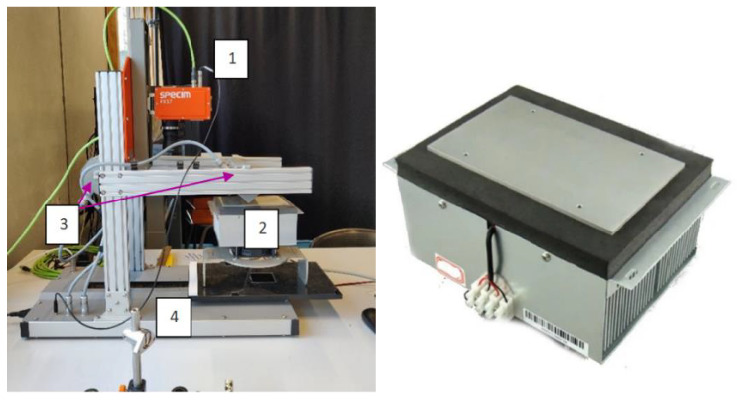
(**Left**): The complete measurement setup, with the hyperspectral camera (1), translation station (4), halogen lamp illumination (3), and TEC (2). (**Right**): The thermoelectric cooler.

To create ice formation in the lab, we utilized a thermoelectric cooler (TEC), specifically a Wakefield-Vette TEMA-AP-80-24 model (Wakefield Thermal, Nashua, NH, USA) (see Figure 1). This device, operating on the Peltier effect, efficiently transfers heat by applying voltage to two different terminals connected by a semiconductor. This setup offers rapid and flexible testing compared with traditional methods like freezers or climate chambers. The TEC operates within a range of −20 °C to 60 °C, with a cooling capacity of 80 W at a 0 °C temperature differential under 30 °C ambient conditions. This allowed us to achieve glaze ice formation from a 2.5 mm water layer in approximately one minute, facilitating repeated measurements on various surface types under different conditions. It is worth noting that while the glaze ice layer inside the rectangular area had a controlled thickness of approximately 2.5 mm, thinner ice layers could not be reliably formed due to capillary effects and surface tension, which prevented the formation of uniform water films on the coated and uncoated aluminum surfaces. However, a much thinner layer of rime ice naturally formed outside the container area due to atmospheric condensation and freezing. This rime ice, although visually subtle, was successfully detected and classified by the trained models, indicating the system’s capability to recognize very thin ice layers as well.

The experiments were conducted using a controlled laboratory setup designed to acquire hyperspectral data from aluminum surfaces under both iced and non-iced conditions. A hyperspectral camera (FX17, Specim, Oulu, Finland) was employed, which captures images in the visible to near-infrared (VNIR) spectrum, ranging from 900 nm to 1700 nm with a spectral resolution of 224 bands. The camera was positioned above a motorized translation stage to enable push-broom scanning of the samples. Uniform illumination was achieved using two halogen lamps, each mounted 25 cm above the surface and angled at 60 degrees relative to the sample plane.

Aluminum samples were selected due to their relevance in aerospace and renewable energy applications. Aluminum plates, selected for their high thermal conductivity, served as test objects. Two plates were coated with different finishes to simulate various surface treatments: one with matte white paint (306 ± 8 μm) and another with black anti-icing finish (274 ± 5 μm). These coatings simulate the diverse materials and finishes used on wind turbine blades. Each plate measures 10 × 15 cm, with a 5 × 8 cm central rectangle in which a small volume of water was poured. We used 10 mL of tap water, achieving a water height of 2.5 mm within the 5 × 8 cm area.

To generate ice under controlled conditions, the TEC was used to cool the aluminum surface beneath a rectangular container filled with water. As the surface temperature dropped, two distinct types of ice formed. First, the water inside the container froze due to direct contact with the cooled aluminum, resulting in a layer of solid ice on the surface. This ice could be classified as either clear or opaque, but for clarity, it is referred to simply as ‘ice’ throughout this thesis. Additionally, the surrounding aluminum surface became cold enough to cause atmospheric water vapor to freeze upon contact, forming a second type of ice: rime ice. Thus, both ice and rime ice were formed simultaneously under these conditions.

Although the primary ice region in our experiments was confined to a rectangular zone to control thickness and facilitate labeling, the classification approach is inherently shape-independent. Because the model operates on pixel-wise spectral signatures, it does not rely on geometric features such as shape or contour. This is further demonstrated by its ability to accurately detect and classify rime ice regions that formed naturally outside the rectangular area, which were irregular in shape and not explicitly part of the labeled training set. These results suggest that the method generalizes well to non-rectangular ice-covered surfaces, supporting its applicability to more complex geometries in practical settings.

The measurement setup is shown in Figure 1.

### 3.2. Hyperspectral Data Acquisition

Hyperspectral image cubes were acquired by translating the samples under the fixed camera to collect a full spatial–spectral dataset. Each hyperspectral cube represents a 2D spatial image with 224 spectral bands per pixel. Reference white tiles were used for calibration, and dark current correction was applied. Reflectance values were normalized with respect to the white reference to eliminate variability due to lighting or sensor inconsistencies [26].

For each surface, data were collected under both dry (non-iced) and iced conditions. Regions of interest (ROIs) were selected from visibly uniform areas with and without icing. These ROIs were used to extract pixel-wise spectral signatures for subsequent classification.

### 3.3. Preprocessing and Feature Selection

A Savitzky–Golay filter was applied to smooth the reflectance spectra and suppress high-frequency noise [27].

**Figure 2:**
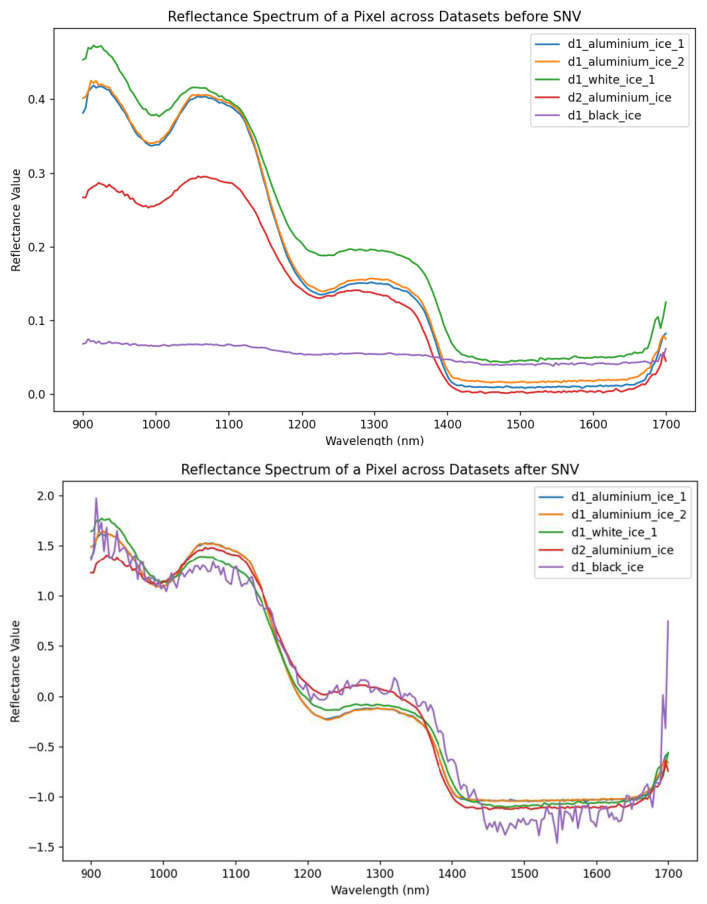
Reflectance spectra across datasets before (**top**) and after (**bottom**) applying Standard Normal Variate (SNV) transformation. The graph shows the reflectance values plotted against wavelengths, with different lines representing various coatings and different datasets (arbitrary selection).

Spectra were then normalized to unit range scaling. Data preprocessing involved the Standard Normal Variate (SNV) method. SNV adjusts each pixel’s intensity based on the mean and standard deviation of its spectral characteristics, facilitating accurate comparisons and analyses across datasets [28]. The formula for SNV is:

(1) $$SNV\left(x_{\lambda }\right)=\frac{x_{\lambda }-\bar{x}}{SD}$$

where λ is the wavelength, $x_{\lambda }$ is the spectral value at the wavelength λ, $\bar{x}$ is the mean spectral value across wavelengths for a given pixel, and SD is the corresponding standard deviation. Figure 2 shows the effect of SNV preprocessing. Prior to SNV correction, there was substantial variation in reflectance values across coatings, with lighter coatings (e.g., white) reflecting more light and darker coatings (e.g., black) reflecting less. After SNV transformation, the absolute differences in reflectance are minimized, potentially reducing the influence of the different coatings. This standardization enhances the comparability of spectral shapes across images, which is particularly beneficial for machine learning-based classification. Notably, the SNV-corrected spectrum of the black-coated surface exhibits more noise than the others, which may affect model performance in this case.

In order to improve classification performance and reduce the risk of overfitting due to the high dimensionality of hyperspectral data, a feature selection step was performed prior to training the models. This process aimed to identify a reduced subset of wavelengths that provided the highest discriminative power for distinguishing between the three classes: no ice, glaze ice, and rime ice. First, the mean reflectance spectra were computed separately for each class using representative samples from the training dataset. These spectra were then compared to identify regions with significant spectral differences between classes. In addition, the standard deviation and inter-class variance across the full spectral range were analyzed to quantify class separability as a function of wavelength.

Based on this analysis, 8 wavelengths were selected that showed the most distinct reflectance patterns among the classes. The selection was guided by visual inspection of spectral contrast, supported by statistical measures of spectral dispersion. These wavelengths likely correspond to specific absorption features or scattering effects associated with the presence and structure of ice, including variations due to surface coatings. By restricting the input feature space to this optimized subset, the classification models could be trained more efficiently and with improved robustness, especially in the presence of limited labeled data.

This approach is consistent with prior work in hyperspectral analysis, where reducing the dimensionality through targeted wavelength selection is known to enhance model interpretability and generalization. Future studies may complement this manual approach with automated feature selection algorithms or integrate it with band selection strategies tailored to real-time implementation.

### 3.4. Machine Learning and Classification Models

This study applies supervised learning, which relies on a labeled dataset for training. The dataset comprises 24 hyperspectral images of an uncoated aluminum test plate, with eight of these images containing visible ice. In each image, square regions of interest are selected around predefined coordinates. The size of these regions is adjusted based on the spatial characteristics of the image to ensure optimal capture of relevant surface features. The hyperspectral data from each selected region are then extracted and reshaped from three-dimensional arrays (height × width × spectral bands) into two-dimensional arrays (combining the width and height), making them more suitable for analysis and machine learning input. Each data subset is labeled according to the presence or absence of ice, resulting in binary labels: ‘positive’ for ice and ‘negative’ for no ice. These labeled subsets are compiled into feature and label arrays, forming the final training dataset. In total, this process yields 7832 labeled data points, which are used to train multiple machine learning models. The goal is for these models to generalize well, enabling accurate ice detection across different surface types and under varying environmental conditions.

The following supervised learning algorithms were implemented:

- Random Forest (RF): An ensemble method that provides band importance scores. Random Forest (RF), an ensemble classification technique, was developed by Breiman in 2001 [29]. It combines bagging and random feature selection, using multiple decision trees to form a forest. Each tree is trained on a distinct subset of data through random sampling with replacement (bootstrap). The results are aggregated into a majority decision [30]. RF’s random feature selection at each decision point reduces tree correlation, enhancing model robustness and efficiency. This technique is widely used for both classification and regression problems across diverse applications [30,31].
- Support Vector Machine (SVM): Optimized for high-dimensional data. Support Vector Machines (SVMs) are supervised learning algorithms for classification and regression tasks [32]. They construct hyperplanes in high-dimensional spaces to separate data into classes, handling both binary and multiclass operations through methods like one-against-one and one-against-rest. SVM maximizes the margin between classes and the decision boundary, with support vectors being the critical data points closest to the hyperplane. The penalty parameter (*C*) balances training error and decision boundary simplicity, helping manage outliers and noise [33]. For non-linear separable data, SVM employs kernel functions (e.g., polynomial, radial basis function, sigmoid) to transform data into higher-dimensional spaces, enabling linear separation. SVMs are effective in high-dimensional spaces and versatile with various kernel functions for complex datasets.

Both Support Vector Machines (SVMs) and Random Forests (RFs) are widely used and high-performing classifiers for hyperspectral image analysis. SVMs are particularly well suited for high-dimensional data with limited labeled samples as they construct optimal decision boundaries through kernel-based learning. They often outperform other methods in homogeneous or small training datasets, achieving overall accuracies up to 95.3% in forest and land cover applications [34,35]. In contrast, RF classifiers are advantageous when dealing with large, heterogeneous datasets due to their robustness to noise, scalability, and lower sensitivity to hyperparameters. Their classification accuracies in hyperspectral settings typically range from 62% to 96%, with the highest results being achieved in complex agricultural and environmental monitoring tasks [36,37]. Several studies emphasize that SVM may slightly outperform RF in accuracy—especially after feature selection or dimensionality reduction [38]—while RF is often computationally more efficient (e.g., training time of 3 min for RF vs. 16 min for SVM in Burai et al. [38]). In this work, both models were used to allow cross-validation of classification outcomes and to leverage their complementary strengths: SVM’s robustness in high-dimensional, low-sample regimes and RF’s efficiency and interpretability across different surface coatings and ice types.

The Support Vector Machine (SVM) and Random Forest (RF) methods were implemented using the Sklearn library in Python 3.12. For the SVM, the SVC implementation with an RBF kernel was selected. Instead of conducting a grid search to optimize parameters, we manually chose values to mitigate overfitting on the training data. The parameters were set to C=1 (error penalty) and γ=0.07 (kernel coefficient). A lower gamma was preferred to broaden the influence of each training data point, helping the model generalize patterns across larger dataset segments. This approach enhances the model’s ability to detect ice under various coatings and conditions, as opposed to focusing on specific features from the training data. For the RF model, different parameters were evaluated, but the default parameters were retained as they performed optimally. It is important to note that the training dataset for both algorithms consisted solely of uncoated aluminum plates. This choice was made to evaluate the models’ ability to generalize ice detection to different coated surfaces during the validation phase.

A wide range of machine learning algorithms has been applied to hyperspectral imaging (HSI) classification tasks, each with strengths and limitations. Among the classical approaches, Support Vector Machines (SVMs) and Random Forests (RFs) remain among the most commonly used due to their effectiveness in high-dimensional feature spaces and strong performance with relatively small training sets. SVM is particularly suited for separating classes in high-dimensional spectral data through the use of kernel functions, while RF excels in handling nonlinearity, noise, and imbalanced datasets. Both methods are computationally efficient and offer good generalization, making them attractive for practical applications in industrial or field settings. Other traditional algorithms such as k-Nearest Neighbors (kNN), Decision Trees, and Linear Discriminant Analysis (LDA) have also been employed but generally deliver lower accuracy or are more sensitive to data variability. More recently, ensemble methods such as Gradient Boosting (e.g., XGBoost, LightGBM) have shown promise, though they typically require more extensive tuning and offer less interpretability. Deep learning models—especially Convolutional Neural Networks (CNNs), 3D-CNNs, and hybrid spectral-spatial architectures—have demonstrated excellent performance in many HSI benchmarks. These models are particularly effective when both spatial and spectral information can be leveraged jointly. However, such models are data-hungry and computationally intensive and may not be optimal when the classification task is primarily spectral and training data are limited.

The recent literature confirms that both classical machine learning techniques—such as Support Vector Machines (SVMs) and Random Forests (RFs)—and deep learning models like Convolutional Neural Networks (CNNs) can yield high classification accuracy for hyperspectral data, with CNNs generally achieving superior results when spatial–spectral features and large annotated datasets are available. However, SVM and RF remain highly competitive, particularly in pixel-wise spectral classification tasks with limited data. For example, Hasan et al. [39] report an overall accuracy of 98.84% using SVM with a radial basis function kernel, surpassing CNN (94.01%) on the same dataset. In another study by Guidici and Clark [40], SVM and a one-dimensional CNN both achieved 90% accuracy, significantly outperforming RF. While CNNs are more powerful for end-to-end spatial–spectral modeling [41,42], they require large datasets, data augmentation, and high computational cost. For applications like ours—focused on spectral signatures without significant spatial structure—SVM and RF offer strong accuracy, lower computational demand, and faster deployment, making them particularly suitable. Hybrid methods (e.g., CNN-SVM [43], HybridSN [44]) may further improve performance, but at the cost of added complexity.

In the context of our study, where pixel-wise classification is based on spectral signatures and the amount of labeled data is constrained, SVM and RF provide a reliable and well-established foundation. Their performance in previous HSI studies and their suitability for generalization across coated and uncoated surfaces make them ideal candidates for evaluating the feasibility of hyperspectral ice detection. Future work will explore the potential of deep learning models and more complex ensembles as larger annotated datasets become available.

In this study, no data augmentation techniques such as image rotation, stretching, or deformation were applied. This is because the classification task was based on pixel-wise spectral signatures extracted from hyperspectral images, independent of spatial context. Unlike in conventional RGB or spatial–spectral classification tasks where geometric augmentations enhance spatial invariance, such transformations are not meaningful for one-dimensional spectral data and may distort the physical properties of the spectra. However, if future approaches incorporate spatial–spectral deep learning (e.g., CNNs), data augmentation may become a relevant strategy, particularly to mitigate class imbalance and improve generalization on more complex scenes.

### 3.5. Evaluation Metrics

The performance of each model was evaluated using standard classification metrics: accuracy, precision, recall, and F1-score [24]. In this study, we employed commonly used metrics—accuracy, precision, recall, and F1-score—to assess the performance of the classifiers across the three classes (no ice, rime ice, glaze ice). These metrics offer interpretable, class-wise insights and are well suited to our balanced dataset. Although additional performance indicators such as the area under the ROC curve (AUC) are valuable in binary or imbalanced scenarios, their extension to multiclass classification requires one-vs-rest decomposition or averaging strategies (macro- or micro-AUC), which may not offer additional discriminative power in our balanced setting. Nonetheless, we acknowledge the usefulness of AUC in future work, particularly if more subtle class imbalances arise or for tasks involving anomaly or rare-class detection.

After training the machine learning models, it is crucial to validate them to assess their performance. This process involves testing the models with validation images, specific images not seen by the model during training. In this research, the validation is conducted using three distinct datasets: uncoated, white-coated, and black-coated datasets. Each dataset includes three validation images, labeled as A1, A2, and A3 for the uncoated dataset; W1, W2, and W3 for the white-coated dataset; and B1, B2, and B3 for the black-coated dataset. Accompanying each validation image is a corresponding ‘ground truth’ image, which contains manually marked zones representing different classes, such as ice. These ground truth images serve as benchmarks, illustrating what the model should ideally predict and providing a basis for comparison against the model’s actual predictions.

In total, 24 hyperspectral images were acquired from the uncoated aluminum surface, of which 15 images—comprising both iced and non-iced regions—were used for training the models. The remaining 9 images were used for validation and included 3 images each from uncoated, white-coated, and black-coated surfaces. Importantly, the coatings used in the validation phase were not included in the training set. This approach allowed us to assess the ability of the models to generalize to unseen surface types, simulating real-world variability in material finishes.

## 4. Results

The results section is structured into three parts:

- Section 4.1: This section focuses on validating the two machine learning models (SVM and RF) by evaluating each coating type separately and then providing a comprehensive evaluation across all coatings.
- Section 4.2: This section examines the models’ performance when fewer spectral bands are utilized, assessing the impact on detection accuracy for all coatings.
- Section 4.3: This section assesses a multiclass Random Forest model, evaluating its capability to detect and differentiate various types of ice.

### 4.1. Classification Performance Using Full-Spectrum Data

**Figure 3:**
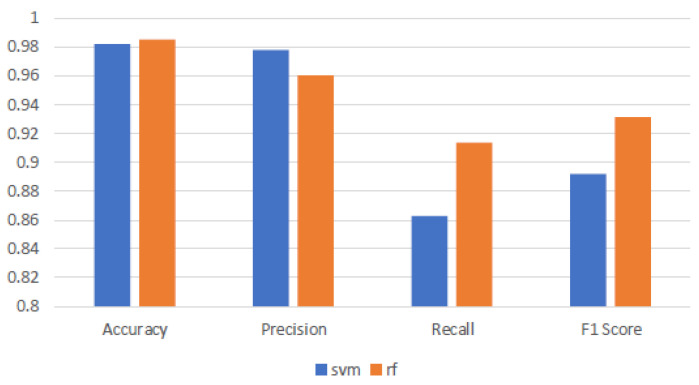
Comparison of SVM and RF models across all the validation images. This bar graph displays the average performance metrics, accuracy, precision, recall, and F1-score, for Support Vector Machine and Random Forest models.

Table 1, Table 2 and Table 3 illustrate the SVM model’s high precision across all surfaces, averaging 97.80%, demonstrating a high degree of predictive accuracy. However, the F1-score significantly varies it remains high on uncoated aluminum and white coatings but drops to an average of 70.12% on black coatings. This results in an overall average across all coatings of 89.15%. The stark drop in performance is primarily due to a dramatic decline in recall from perfect on more reflective surfaces to just 59.06% on black coatings. This decline in recall on black-coated surfaces critically undermines the F1-score, highlighting SVM’s struggle to balance detection sensitivity with precision with more challenging data. Conversely, the RF model, while slightly trailing behind SVM with an average precision of 96.02%, displays superior recall capabilities across surfaces, averaging 91.33%. Its F1-scores, notably higher than those of SVM, average 87.93% across all surfaces, particularly excelling on those where SVM struggles. This demonstrates RF’s robustness and its ability to maintain a better balance between precision and recall, especially with more challenging data. Figure 3 shows that both the SVM and RF models exhibit high accuracy and precision across various surface coatings, demonstrating their robust capability in making correct classifications and minimizing false positives. However, comparing the recall and F1-scores of the models reveals a decline in performance for both models, with SVM experiencing a more significant drop. This underperformance is attributed to the challenges posed by the black-coated surfaces, likely caused by the increased absorbance. Such noise complicates the models’ ability to consistently identify true positives, impacting SVM more severely than RF. This effect is reflected in the lower F1-scores for SVM, highlighting its struggle to maintain a balance between precision and recall in the presence of noisy data. The robust recall capability of RF ensures that RF’s F1-scores remain high, affirming its ability to effectively balance precision and recall, making it particularly valuable in applications where detecting every instance of ice presence is crucial.

**Figure 4:**
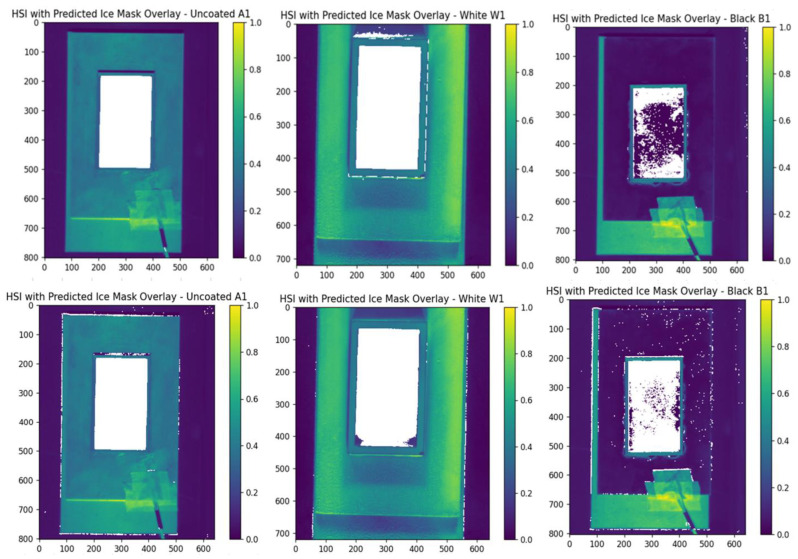
Prediction result of the SVM (**top**) and RF (**bottom**) model on the different coated samples A1 (uncoated), W1 (white coating), and B1 (black coating). The white area represents the predicted ice location. The ground truth ice location is the complete rectangular box in the middle.

Figure 4 presents the 2D ice detection results for datasets A1, W1, and B1 using both SVM and RF classifiers. White regions indicate pixels predicted as ice. The results show that both models perform noticeably worse on the black-coated sample (B1). In this case, several ice-covered pixels are not correctly identified, and the RF model additionally misclassifies non-ice pixels outside the rectangular region as ice.

### 4.2. Performance Evaluation with Spectral Band Reduction

Hyperspectral imaging in this study captures 224 spectral bands per image, leading to large data volumes requiring significant storage and processing time. To address this, we evaluate the impact of using fewer spectral bands on data demands, processing time, and model accuracy in detecting ice. Two reduced band ranges are considered:

- First 65% of bands: Bands 1 to 146 (900 to ±1450 nm). The upper limit of this range, around 1450 nm, corresponds to a major absorption peak of both water and ice, resulting from multiple overtone and combination vibrational modes.
- First 50% of bands: Bands 1 to 112 (900 to ±1300 nm).

New SVM and RF models were trained for each band range. Training data and settings remained consistent with the full-band models, except for an SVM parameter adjustment. The gamma parameter was increased from 0.07 (full-band model) to 0.1 for both reduced band ranges to improve ice classification accuracy. These new models were validated using the same validation sets as before.

**Figure 5:**
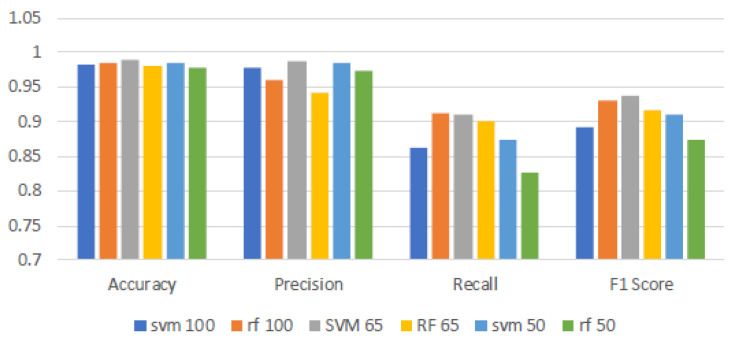
Results of SVM and RF models for different band levels. The band levels are defined as follows: 100% (all bands used), 65% (bands 1 to 146 used), and 50% (bands 1 to 112 used).

At the 100% band level, SVM demonstrates strong performance with an average accuracy of around 98% (see Figure 5). It shows robust precision, also close to 98% across all coatings. However, while recall (99%) and F1-score (99% and 98%) are high for the uncoated and white-coated surfaces, the recall for the black-coated surface drops significantly, averaging around 59%. This impacts the overall F1-score, reducing it to approximately 89%. This indicates that while SVM is highly precise, its ability to correctly identify all relevant instances diminishes, particularly on certain coatings. When 65% of the bands are used, SVM maintains a high level of performance. Most metrics improve across the different coatings. Notably, the recall for the black-coated surface improves to about 73%, while precision remains high, even improving to 99.22%, with an average overall recall of 91.01% and precision of 98.72%, suggesting that the model becomes better at correctly capturing relevant instances of ice despite using fewer bands. With further reduction to 50% bands, SVM continues to exhibit high metrics for the uncoated and white-coated surfaces, with accuracy (95.89%) and precision (98.88%) remaining fairly similar to the other band ranges. However, the recall for the black-coated surface drops significantly to around 63% compared with that of the 65% band range, which affects the F1-score, lowering the overall average to about 90.91%. This indicates that while performance drops compared with the 65% bands, it is still an improvement compared with using all 224 bands.

RF also exhibits robust performance across different band ranges. At the 100% band level, RF achieves high accuracy around 98%, with robust precision close to 96%. It demonstrates strong recall capabilities, averaging around 91.33%, leading to an F1-score of 93.16%. This suggests that RF is proficient in both identifying relevant instances and maintaining precision. At the 65% band range, RF’s performance for the uncoated surface remains high and stable, with an average F1-score of around 98%. However, the performance for the white-coated surface drops significantly, with recall at 90.17%, precision at 94.20%, and an F1-score of 92.10%. For the black-coated surface, what it loses in precision it gains in recall, resulting in an F1-score of 84.84%, similar to when all bands are used. When the bands are reduced further to 50%, RF maintains high metrics for the uncoated and white-coated surfaces. The reduction in bands does not negatively impact these metrics, with the metrics for the white coating being very similar to those when all bands are used. The metrics for the uncoated surface even improve, with precision at 97.36% and an F1-score of 98.64%. However, performance for the black-coated surface decreases, with recall dropping to 52.44% and an F1-score of 66.39%. Based on Figure 3, it can be concluded that for SVM, accuracy and precision remain constant and robust when reducing the number of bands. Both recall and F1-score for SVM increase when the number of bands is reduced to 65% and 50%, with the model performing optimally at 65% bands. In contrast, the RF model does not benefit from reducing the number of bands. Although RF gains in recall, it loses in precision, performing best at 100% bands. The most significant factor contributing to the worsening results when reducing the number of bands for both models is the performance on black-coated surfaces. These findings underline the potential to optimize hyperspectral imaging by tailoring the spectral band utilization to specific model characteristics and application needs, enhancing both data processing efficiency and detection accuracy.

### 4.3. Classification of Ice Types Across Surface Conditions

This section presents the validation of a multiclass ice detection approach aimed at identifying and distinguishing between different types of ice. Such capability is important, as various ice forms can have differing impacts on turbine performance and operational safety.

During the experiments, cooling water in the rectangular container with a Peltier element caused the aluminum surface to reach temperatures low enough for ice formation. This process resulted in two types of ice: one formed from the freezing of liquid water inside the container, referred to here as ‘ice’ (though it could be further classified as clear or opaque ice), and a second layer, formed from atmospheric water vapor condensing and freezing on the surrounding surface, identifiable as rime ice.

To enable multiclass detection, the Random Forest (RF) model was retrained using the same dataset previously used for binary classification. The main difference was the relabeling of certain data points: instead of using ‘false’ for non-ice pixels, these were reclassified as ‘rime’ to reflect the presence of rime ice. Only the RF algorithm was used for this multiclass validation. Although Support Vector Machines can be extended to multiclass tasks, the SVM model failed to generalize adequately across different ice types with the same training data, and was therefore excluded from further evaluation. Validation was conducted on the same three datasets used in earlier binary classification experiments.

**Figure 6:**
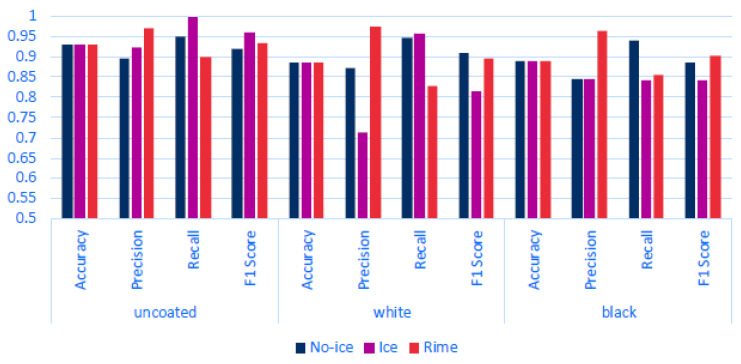
Performance metrics for multiclass classification on different coatings. This bar graph illustrates the classification results for three classes, no ice, ice, and rime, across three different surface coatings: uncoated, white, and black. Each set of bars shows the accuracy, precision, recall, and F1-score for the respective surface and class, highlighting the effectiveness of the classification approach under varying conditions.

**Figure 7:**
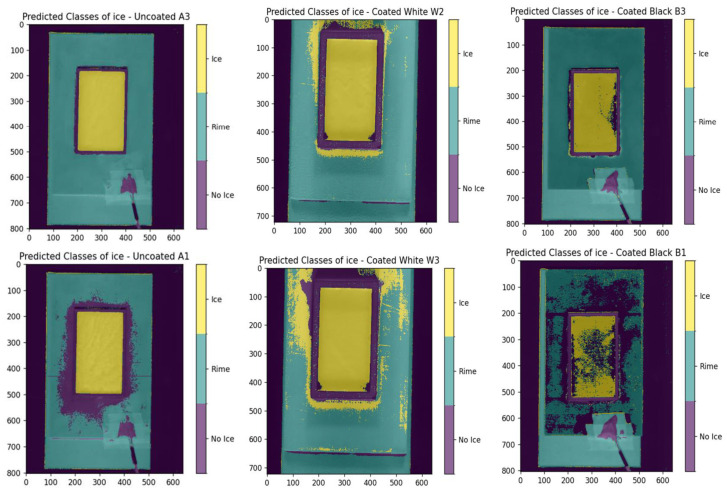
Prediction results of ice formation on uncoated (A), white-coated (W), and black-coated (B) surfaces. The color-coded maps indicate regions classified as ice, rime, or no ice. The top row displays examples where the classification performed well, while the bottom row shows cases with poor classification accuracy.

Assessing the performance of a Random Forest (RF) multiclass model across different datasets using Figure 6 and Figure 7 the following can be observed: On the uncoated dataset, the RF model demonstrates robust performance, achieving a high accuracy rate of 93.00%. This underscores the model’s effectiveness in accurately detecting various classes. Specifically, the ‘ice’ and ‘rime’ classes show impressive precision rates of 92.33% and 97.05%, respectively, indicating that the model’s predictions for these types of ice are highly reliable. Moreover, the ‘ice’ class exhibits nearly perfect recall at 99.77%, effectively identifying almost all instances of this class. However, the ‘rime’ class experiences a drop in recall to approximately 90%. This decrease is attributed to certain instances, such as those illustrated in Figure 7 in the bottom left, where the model struggles to detect all occurrences of ‘rime’ around the edges of the rectangle. Overall, the model proves to be quite capable on uncoated surfaces, adeptly detecting and distinguishing between different ice types, as evidenced by the strong F1-scores (92.03% for no ice, 95.91% for ice, and 93.13% for rime) and by the bottom middle of Figure 7. In the white-coated dataset, there is a noticeable decline in overall accuracy to 88.40%. This reduction marks the initial indication of the potential impact that white coating has on the model’s ability to accurately distinguish between classes. For the ‘ice’ class, precision significantly decreases to 71.41%, though it still maintains a high recall. Conversely, the ‘rime’ class exhibits high precision at 97.51% but a lower recall at 82.91%. This discrepancy suggests a tendency for the model to overpredict the presence of the ‘ice’ class, leading to a higher rate of false positives, as illustrated in the bottom right of Figure 7. While the model is capable of detecting the presence of ice, it struggles more with differentiating between the two ice classes. In analyzing the metrics for the black-coated dataset, it is important to consider the known issue of data noise. Despite this, the ‘rime’ class performs very well, achieving a high precision of 96.30% and a decent recall, resulting in a relatively good F1-score of 90.13%. On the other hand, the ‘ice’ class shows underperformance in both recall (84.17%) and precision (84.49%). The lower recall indicates that not all instances of the ice class are detected, as seen in the bottom left of Figure 7. The ‘rime’ class experiences a similar recall issue. The lower precision for the ‘ice’ class is primarily due to the model’s tendency to misclassify the edges of objects as ‘ice’, which is evident in Figure 6 and Figure 7.Considering both the previous and current validation results, we can conclude that the model effectively differentiates between various ice classes on uncoated and black-coated surfaces. On uncoated surfaces, the model can detect almost all instances of ice, demonstrating its high sensitivity. Similarly, on black-coated surfaces, the model identifies most ice instances, although its performance is contingent upon the quality of the data. Conversely, while the model on white-coated surfaces can detect most of the ice, it struggles more with accurately differentiating between the ice classes, often overpredicting the presence of the ‘ice’ class.

## 5. Conclusions

This study demonstrated the potential of hyperspectral imaging (HSI) combined with machine learning for reliable ice detection on coated and uncoated surfaces representative of wind turbine blades. Two classification algorithms, Support Vector Machine (SVM) and Random Forest (RF), were trained on data from uncoated aluminum and validated on surfaces with different coatings.

The results show that both models generalize well to unseen surface coatings, with consistently high performance on white and uncoated samples. Performance, however, was notably reduced on black-coated surfaces, likely due to increased absorbance of the coating. Despite this, the models achieved high accuracy overall, confirming the effectiveness of combining HSI and machine learning for surface ice detection.

The study also explored spectral band reduction to evaluate its effect on classification accuracy. SVM performed best with a reduced range (65% of the original wavelength range), while RF achieved optimal results using the full spectrum (bands 1–224). These findings suggest that tailored spectral selection can improve computational efficiency without significantly compromising performance, depending on the model used.

Furthermore, a multiclass classification approach using RF demonstrated the ability to differentiate between rime and glaze ice. This capability is essential for context-aware mitigation strategies in safety-critical applications like wind energy.

The multiclass classification framework presented in this study demonstrates the feasibility of distinguishing between no ice, glaze ice, and rime ice using hyperspectral imaging combined with classical machine learning models. This has clear potential for real-world applications such as automated aircraft surface inspection, wind turbine blade monitoring, and power line safety checks—where the type of ice affects both risk and appropriate mitigation strategies. The ability to differentiate between ice types could improve early-warning systems, maintenance scheduling, and de-icing procedures. However, several limitations remain. First, environmental variability (e.g., illumination, temperature, or contamination) may affect spectral signatures, potentially requiring retraining or adaptation. Second, while the current model generalizes to unseen surface coatings, performance on more complex geometries or textured materials has not yet been validated. Finally, real-time deployment would require optimization of acquisition speed and onboard processing, as well as integration with decision-making systems.

Future research should address the current limitations and further improve the robustness and applicability of the proposed method. Specifically, domain adaptation techniques should be explored to enable generalization across diverse environmental and material conditions. Challenges posed by dark coatings—such as lower reflectance and higher signal noise—should be addressed through improved preprocessing pipelines or model training strategies that incorporate noisy or low-SNR data. Additionally, future work should investigate the potential of deep learning architectures, including spatial–spectral Convolutional Neural Networks (e.g., 2D/3D CNNs), which may enhance performance by capturing both spectral and structural information, especially for detecting partial or thin ice layers and estimating ice thickness. Real-world deployment will also require on-site validation using wind turbine blades or other critical infrastructure under operational conditions. These tests would confirm the method’s robustness in varying illumination, temperature, and weather conditions. Furthermore, integrating complementary sensing modalities, such as thermal imaging or LiDAR, may strengthen detection in complex or low-visibility environments. Finally, real-time implementation on embedded or mobile platforms (e.g., drones, robotic arms) should be pursued by optimizing the acquisition and inference pipeline to meet the practical constraints of field operation.

## Figures and Tables

**Table 1:** Performance metrics (accuracy, precision, recall, F1-score) for datasets A1, A2, and A3 and the average (uncoated samples) for the SVM and RF methods.

Data	Accuracy	Precision	Recall	F1-Score
SVM				
A1	0.9979	0.9824	0.9990	0.9906
A2	0.9978	0.9807	1.0000	0.9902
A3	0.9978	0.9805	1.0000	0.9901
Avg	0.9978	0.9810	0.9997	0.9903
RF				
A1	0.9938	0.9472	1.0000	0.9729
A2	0.9941	0.9490	1.0000	0.9738
A3	0.9941	0.9494	1.0000	0.9740
Avg	0.9940	0.9485	1.0000	0.9736

**Table 2:** Performance metrics (accuracy, precision, recall, F1-score) for datasets W1, W2, and W3 (samples with white coating) and the average for the SVM and RF methods.

Data	Accuracy	Precision	Recall	F1-Score
SVM				
W1	0.9947	0.9675	1.0000	0.9834
W2	0.9948	0.9681	1.0000	0.9837
W3	0.9942	0.9680	0.9961	0.9818
Avg	0.9946	0.9679	0.9987	0.9830
RF				
W1	0.9924	0.9931	0.9581	0.9753
W2	0.9924	0.9920	0.9594	0.9754
W3	0.9904	0.9933	0.9457	0.9689
Avg	0.9917	0.9928	0.9544	0.9732

**Table 3:** Performance metrics (accuracy, precision, recall, F1-score) for datasets B1, B2, and B3 (samples with black coating) and the average for the SVM and RF methods.

Data	Accuracy	Precision	Recall	F1-Score
SVM				
B1	0.9157	0.9750	0.2356	0.3795
B2	0.9638	0.9904	0.6788	0.8055
B3	0.9833	0.9891	0.8575	0.9186
Avg	0.9543	0.9848	0.5906	0.7012
RF				
B1	0.9457	0.9094	0.5597	0.6929
B2	0.9833	0.9540	0.8915	0.9217
B3	0.9848	0.9541	0.9055	0.9292
Avg	0.9713	0.9392	0.7856	0.8479

## Data Availability

The data that support the findings of this study are available from the corresponding author upon reasonable request.
